# The Cajal School in the Peripheral Nervous System: The Transcendent Contributions of Fernando de Castro on the Microscopic Structure of Sensory and Autonomic Motor Ganglia

**DOI:** 10.3389/fnana.2016.00043

**Published:** 2016-04-20

**Authors:** Fernando de Castro

**Affiliations:** Grupo de Neurobiología del Desarrollo-GNDe, Instituto Cajal-CSICMadrid, Spain

**Keywords:** Nobel Prize, history of neuroscience, Santiago Ramón y Cajal, spanish neurohistological school, superior cervical ganglion, development, synapse, chemoreceptors

## Abstract

The fine structure of the autonomic nervous system was largely unknown at the beginning of the second decade of the 20th century. Although relatively anatomists and histologists had studied the subject, even the assays by the great Russian histologist Alexander Dogiel and the Spanish Nobel Prize laureate, Santiago Ramón y Cajal, were incomplete. In a time which witnessed fundamental discoveries by Langley, Loewi and Dale on the physiology of the autonomic nervous system, both reputed researchers entrusted one of their outstanding disciples to the challenge to further investigate autonomic structures: the Russian B.I. Lawrentjew and the Spanish Fernando de Castro developed new technical approaches with spectacular results. In the mid of the 1920’s, both young neuroscientists were worldwide recognized as the top experts in the field. In the present work we describe the main discoveries by Fernando de Castro in those years regarding the structure of sympathetic and sensory ganglia, the organization of the synaptic contacts in these ganglia, and the nature of their innervation, later materialized in their respective chapters, personally invited by the editor, in Wilder Penfield’s famous textbook on Neurology and the Nervous System. Most of these discoveries remain fully alive today.

## Introduction

Today it is common knowledge that in Vertebrates an autonomic (or vegetative) nervous system governs the visceral components of the body and controls the internal environment in close integration with the somatic nervous system. The autonomic nervous system has sensory and motor ganglia, and the latter can belong to the sympathetic or parasympathetic subdivisions. In addition there are two related but semi-independent systems in the heart and the enteric system (Standring, 2008). At the beginning of the 20th century, although knowledge of the general microscopic structure of the nervous system was accumulating fast due to, among others, the capital contributions by Santiago Ramón y Cajal (1852–1934), several neural structures remained poorly understood. Amongst these were the relatively small groups of neural cells forming the sensory and autonomic ganglia, all of them external to the mechanical protection offered by the skull and vertebrae lining the vertebral canal. The concept of the autonomic nervous system had been proposed by the British neurophysiologist John Langley (1852–1925):

“I propose the term “autonomic nervous system” for the sympathetic system and the allied nervous system of the cranial and sacral nerves and for the local nervous system of the gut” (Langley, 1898).

With this concept, Langley modified previous descriptions by Christian Bell (“vegetative nervous system”) and François-Xavier Bichat (“ganglionic nervous system”). Langley reserved Winslow’s “sympathetic nervous system” to those ganglia positioned closely to the thoracic and lumbar spinal cord, and he coined that of “parasympathetic” for the cranial and sacral ganglia involved in the visceral innervation. This was maybe the first global conclusion after experimental work started around 1889 when blocking the of peripheral ganglia with nicotine had made it possible to distinguish between preganglionic fibers projecting to ganglionic cells and other fibers surpassing ganglia to innervate organs (Langley and Dickenson, 1889). Langley proposed that it is a single sympathetic cell that connects the CNS and the final effector organ. He considered each ganglion as a switching station and classified efferent nerves as “preganglionic” or “postganglionic”. In a series of research articles during the 1890s, Langley and his then young pupil Charles S. Sherrington (1857–1952) established the concept of an “innervation field” by describing the distribution of sympathetic fiber terminals in the skin (for a summary, see Todman, 2008). In 1921, the German physio-biochemist Otto Loewi (1873–1961) published his famous experiment on the beating hearts of frogs (with and without vagus nerve, respectively), which allowed him to propose that a chemical substance (“Vagusstoff”), liberated by nerve terminals, controls the frequency of heart contractions (Loewi, 1921). This ”Vagussoff’ was later identified as acetylcholine by Henry Hallett Dale (1875–1968), who identified also a related molecule in (ortho)sympathetic neurons: adrenaline. Thus, acetylcholine and adrenaline were the first neurotransmitters identified (Dale and Richards, 1927; Dale and Dudley, 1929). It was in the parasympathetic system where the chemical component of synaptic transmission was first recognized (although we know today that there are cases of electrical synapses). For these important discoveries Loewi and Dale received the Nobel Prize in Physiology or Medicine in 1936.

**Figure 1:**
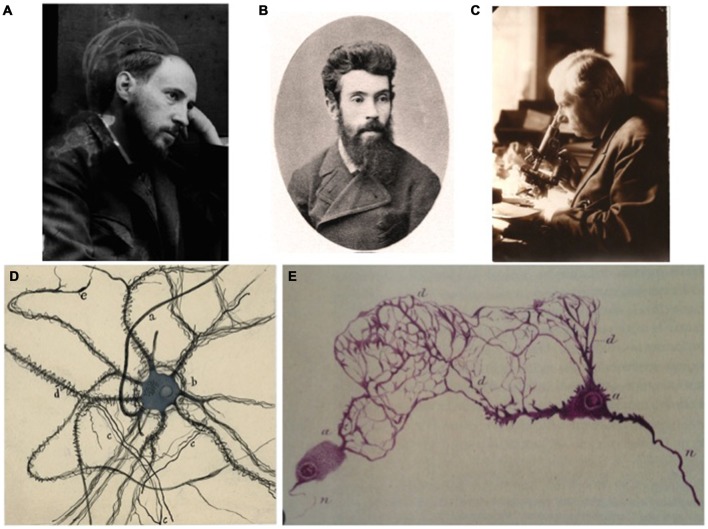
**A tribute to some pioneers of the study of the autonomic nervous system. (A)** Photographic self-portrait of Santiago Ramón y Cajal; his profile is outlined by a chalk sketch of a human brain. **(B)** Portrait of the recognized Russian histologist Alexander Dogiel. **(C)** The Russian neuroscientist Lawrentjew looking at the microscope. **(D)** A detailed drawing from a sympathetic neuron by Santiago Ramón y Cajal, as a good example of the neuronist interpretation of the fine structure of the nervous system. **(E)** Dogiel’s interpretation of neurons from the Auerbach plexus (published in: Dogiel, 1899), a good example of reticularist vision of the organization nervous system **(A–D)** are part of Archive Fernando de Castro.

In spite of all these capital developments in the understanding of the physiology of the peripheral nervous system the study of the fine morphology of the associated anatomical structures and their interconnectivity remained technically and logistically very challenging. Many histologists had tried to resolve the morphological details, among them Alexandre Dogiel, Santiago Ramón y Cajal, Michael von Lenhossèk, Gheorghe Marinescu, Jean Nageotte, Károly Schaeffer, Max Bielschowsky, and Giusseppe Levi, for merely citing the most relevant ones (Figures 1A–C; good reviews on prior works can be found in, respectively, de Castro, 1932a, 1951). Dogiel (1852–1922) was the first identifying different types of neuron in somatosensory, sympathetic and parasympathetic ganglia (Dogiel, 1899). Studying the enteric ganglia with different histological methods (Ehrlich and Golgi methods, respectively), Dogiel described particular cells with short dendrites later named after him. Cajal discovered the stellate cells with long dendrites (Ramón y Cajal, 1899; Figure 1D; “colossal dendrites” in his own words). This is a good example of the complementarity of the different studies in clear (and sane) scientific competitiveness. It is therefore not surprising that the interpretations of all these pioneers differed almost as much as their nationalities or as the animal species studied. In its apogee at that time, the debate between supporters of “neuronism” (i.e., Cajal) and “reticularism” (Figures 1D,E) was even bitter in this particular field because researchers like Kölliker and Dogiel (both cannot generally be considered as reticularists) thought that the interstitial Cajal cells in the gut were fibroblastic while Cajal proposed their neural origin (for a review on this specific topic, see Szentágothai, 1975; García-López et al., 2009).

Although the contributions by Giuseppe Levi (1872–1965) on the sensory ganglia were really remarkable (Levi, 1908), important debates took place on the intraganglionic axon collaterals and on the nature of the “atypical cells” [cells with fenestrated forms, tangled, or with cell processes in balls (terminology of those days)]. This was undoubtedly why Ramón y Cajal entrusted his young pupil Fernando de Castro (1896–1967) to work on the microscopic structure of the sensory ganglia that, with time, would crystallize in a brilliant PhD thesis:

“Hey, guy, here are some badly-known details, for example the interpretation of typical and atypical cells found in the sensory ganglia. We really do not know beyond what is described under experimental conditions. In the human we do not know what exactly the atypical forms are, especially in normal conditions and in young humans” (Gómez-Santos, 1968).

In other words, are the atypical forms of cells observed in the sensory ganglia a fruit of pathological processes affecting ganglia or can they be observed in normal conditions, too?

## De Castro’s First Steps in Science: Structure of the Human Sensory Ganglia

With this commission received from the Maestro (Ramón y Cajal), de Castro started to accumulate material from autopsies. He systematically collected Gasser’s (Vth cranial nerve; somatic sensory) and vagus plexiform ganglia (Xth cranial nerve; autonomic sensory), in order to systematize the findings by his predecessors Cajal and Bielschowsky who had obtained them with neurofibrillary silver impregnations. “Normal” material was obtained from premature human fetuses to young adults (40–45 years-old) died accidentally. Ganglia in pathological cases included specimen obtained post mortem from patients suffering from a large diversity of diseases, ranging from infectious diseases (syphilis, tetanus, tuberculosis, rabies, Kala-azar, etc.) to metastatic cancers, intoxications and alcoholism, osteomalacia, diabetes, hyperthyroidism, amyotrophic lateral sclerosis and traumata. He applied the silver methods of Cajal, Achúcarro and Río-Hortega. De Castro confirmed that monopole neurons are the most abundant cell type, up to 70% of the total cells in the normal sensory ganglia, slightly more than calculated by Cajal and Marinesco and three times more than the number obtained by Levi (de Castro, 1922). This type of cell was even more abundant in pathological conditions, especially in the prenatal samples. Both Levi and de Castro confirmed that the largest of these cells occurred in cervical and lumbar ganglia and within a single ganglion, at its poles (de Castro, 1922). It is remarkable that although Levi and Terni had previously described a relationship between the size of the ganglionic neurons and the volume of peripheral tissue innervated by its axon (Terni, 1914), de Castro briefly cited this observation without intellectual additions (de Castro, 1922) and ignored it in his next chapter on the subject (de Castro, 1932a), maybe due to the fact that these observations had been made in reptiles. A bulk of posterior data in the same sense as those described by the Italian pioneers, including many observations in mammals, resulted in what is known as the “neurotrophic theory” (for a compilation of this, see Purves, 1988). De Castro’s observations confirmed prior descriptions from Cajal, Dogiel and others with respect to the presence of bipolar cells in normal ganglia but he reported that neurons with intraganglionic branches (Dogiel’s type VIII) were only present in pathological circumstances, in open contradiction with Dogiel (Dogiel, 1908; de Castro, 1922). While Ramón y Cajal was the first to describe satellite cells in somatic sensory ganglia, Dogiel hypothesized that they had mesenchymal origins, but it was de Castro with an elegant combination of different histological staining techniques who clearly demonstrated their ectodermal nature and their function as “neuro-neuroglial symbiosis” (de Castro, 1922). De Castro also confirmed previous observations of Cajal and Levi (1908) and as a result, suggested that Dogiel’s type V, VI and VII should be considered as variations of the same cell type (fenestrated cells), present in both normal and pathological conditions (Dogiel, 1908; de Castro, 1922). In all these specific questions, the state of the art remained almost unaltered for at least 10 years (de Castro, 1932a).

In the study of the ganglia obtained from cases with pathological conditions, (de Castro, 1922) appeared involved in a curious debate at that time. Some experts in the field (Dogiel, Michailow, Cajal—initially) assumed that the ball-ended processes arising from ganglia after nerve transection had a trophic function. De Castro leaned towards the alternative view that these balls grew to repair nerves after their destruction (Marinesco, de Castro, Cajal—in a second stage). This alternative view was strongly supported by the *in vitro* regenerative studies performed mainly by Marinesco and Minea (Marinesco and Minea, 1912, 1914). The current perspective on the subject is a concept of molecular differentiation between both trophic and tropic cues in growing and re-growing of axons (Tessier-Lavigne and Goodman, 1996; de Castro, 2003). At the beginning of the 1930s, following the initial descriptions of nerve regeneration by Langley (Langley, 1898, 1900), de Castro undertook complex experiments including reinnervation and crossed anastomosis between autonomic motor fibers and sensory ganglia. He as well studied the behavior *in vitro* of explants from ganglia (de Castro, 1930, 1933, 1934, 1937), but these studies were mostly focused on subjects outside the scope of the current review.

**Figure 2:**
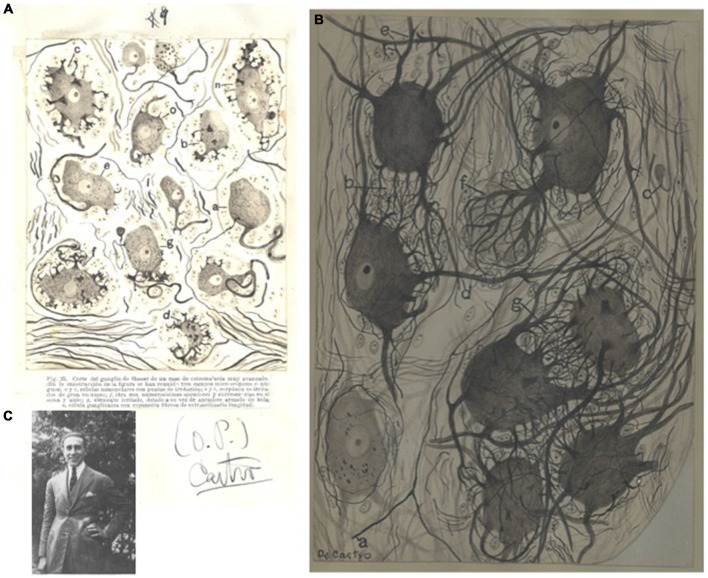
**First works of Fernando de Castro in the structure of the peripheral nervous system. (A)** Image from the original PhD thesis of Fernando de Castro, with his hand-drawn illustrating some pathological forms of neurons from the Gasser’s ganglion from a patient of osteomalacia. The typewritten figure legend (in Spanish) for the defense of the thesis is conserved for the reader, as well as the signature from de Castro at that time. This figure was published in de Castro (1922). **(B)** de Castro’s original hand-sketch of a portion of a sympathetic lumbar ganglion in normal condition (human, 38-year old) originally stained with the Cajal’s method, and illustrating preganglionic (*a*) and intraganglionnic endings (*d*) over dendritic bushes, accesory dendrites forming bushes (*b,g*), a protoplasmic process forming collaterals (*c*) and a pericellular dendritic nest (*f*). This schema was published in de Castro (1923c, 1933). **(C)** Image of a young Fernando de Castro (1922) at his family house in Cercedilla, in the mountains close to Madrid **(A–C)** are part of Archive Fernando de Castro.

In his research on experimental re-innervation and regeneration, de Castro produced several of his most memorable histological preparations and drawings reflecting the diversity of cells and the complex relationships between neurons (Figure 2; de Castro, 1921, 1922). The publication of these intense and meticulous studies had important consequences. Some of them are easily tangible. For instance, de Castro’s PhD thesis, named “Estudio de los ganglios sensitivos del hombre en estado normal y patológico. Formas celulares típicas y atípicas” defended at the Medical School of the Universidad de Madrid (Spain) in 1922 (Figure 2A), obtained the highest possible qualification (“Sobresaliente”) and was 1 year later awarded by the Real Academia Nacional de Medicina with the Rodríguez Abaytúa Prize. But the ultimate award for de Castro’s scientific career was the definitive and full scientific and technical recognition by the Maestro, Santiago Ramón y Cajal. This recognition did not weaken: it would last until the death of Cajal in 1934 and would determine several of the milestones in the scientific trajectory and human life of Fernando de Castro.

## The Second Conquest: The Fine Structure of Autonomic Ganglia

**Figure 3:**
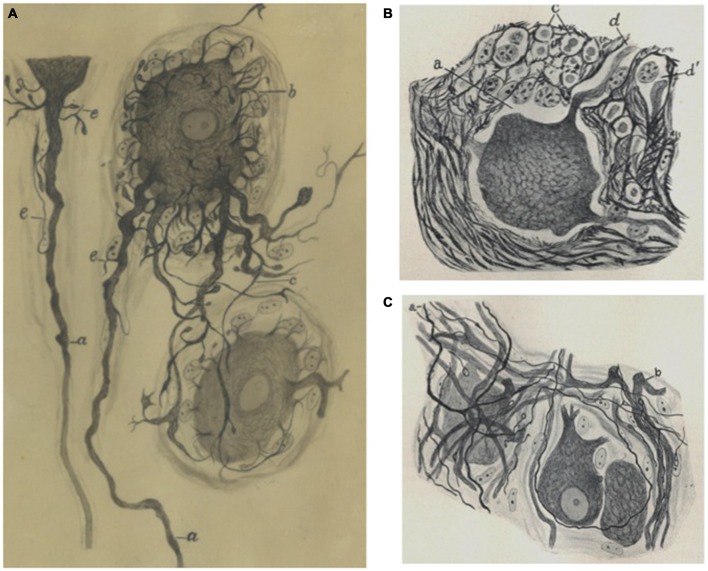
**Sympathetic neurons by Fernando de Castro. (A)** de Castro’s hand-made schematic illustration of sympathetic neurons stained with the Cajal’s method, showing short long (*a* –the axón arises from this dendrite at a distance from the soma, *c*), short dendrites (*b*). This image was published in de Castro (1933). **(B)** Partial view of a sympathetic ganglion (normal condition) of an adult cow (de Castro, 1937). **(C)** Portion of a sympathetic ganglion with regenerated preganglionic fibers (*a*) after a vagus-sympathetic crossed anastomosis (de Castro, 1937; **A)** is part of Archive Fernando de Castro.

Undoubtedly impelled by the success of his research on the histology of the human somatic sensory ganglia, as well as by the evident lack of studies with neurofibrillary methods at that time, Fernando de Castro re-assumed a research line which he briefly explored in the very first years of his scientific career (de Castro, 1916): a serious study of the histology of autonomic ganglia. This research line can be considered as completed with the publication of a monograph and a series of shorter articles which includes maybe the most important comparative study between mammalian species including primates and humans to that date (de Castro, 1923a,b, 1926, 1927), although important morpho-functional observations derive from works mainly devoted to other subjects than sympathetic ganglia (for details about the latter three studies, see below de Castro, 1932a,b, 1937, 1942; de Castro and Herreros, 1945; Figure 3). Together with research by other colleagues (Van Gehuchten, Lenhossek, Retzius, Köelliker, Cajal, Mihailov, etc.), de Castro’s contribution during 25 years of work in this field affirmed that the preganglionic connections wrap in spirals onto ganglionic cells to form the pericellular nests described by Ehrlich in the frog (although the number of these nests are largely lower in mammals; Ehrlich, 1888; de Castro, 1923a,b, 1932b). De Castro also affirmed that these dendritic nests, far from being accidental arrangements, are receptive sites for specific synaptic contacts from preganglionic fibers (de Castro, 1923b). Indeed, the fibers climbing along the dendrites, forming what they called the “receptive plaques”, were pointed by Cajal and de Castro as maybe the most frequent form of intercellular connection in the sympathetic ganglia (de Castro, 1923a,b, 1933, 1951). It was almost a decade after these first descriptions by de Castro that synapses were suggested to be present at the terminal boutons of the preganglionic fibers (de Castro, 1930, 1933; Lawrentjew, 1931, 1934a,b; Kolossow and Sabussow, 1932; Fedorow and Matwejewa, 1935; Bullón Ramirez, 1945; Bullón-Ramirez, 1947). These morphological descriptions contributed to the notion that three main types of neuron can be distinguished in the sympathetic motor ganglia (big, medium size and small neurons, big and small neurons each approximately 25% of the population, and medium size 50%). In each ganglion cells of these three types are intermingled and distributed in an apparent arbitrary way (de Castro, 1932b, 1937, 1950), and each type of preganglionic fiber contacts exclusively one type of ganglionic cell (Billingsley and Ranson, 1918; de Castro, 1923a, 1932a,b, 1937), which coincides with electrophysiological recordings showing four different potential waves in sympathetic ganglia (Bishop and Heinbecker, 1932; Eccles, 1935a,b,c). In this sense, de Castro’s observations on the nature of the axons of Dogiel’s Type II cells confirmed that they project either to other neurons within the same ganglia or in other neighbor ganglia, while they never end in the enteric mucosa. These observations confirmed previous reports (Dogiel, 1899; Ramón y Cajal, 1905; Billingsley and Ranson, 1918; de Castro, 1923b). Thus, the sensory nature of these fibers, as proposed originally by Dogiel, could be discarded. Posterior denervation studies demonstrated that the number of intraganglionic synapses is significantly larger than that of terminal boutons (de Castro and Herreros, 1945). In the latter article the positioning of the synaptic boutons close to astrocytes suggests the presence of what has been described at the turn of the 21st century as “tripartite synapses” (Araque et al., 1999; Perea et al., 2009). Developmental evidence drove de Castro to suggest that the apparent disorder and arbitrary distribution of ganglionic cells derive from germinative centers or spheres disseminated within the ganglia (de Castro, 1923a, 1932a,b). Although they appear in these studies as modest details, de Castro’s mind caught details here that remain uncontested and are still very important for our current perception of the structure and functioning of the nervous system. For example, he clearly stated that the ganglia are literally invaded by mesenchymal structures that lie interposed between the ganglionic neuronal components (somata, dendrites, axons). There always appeared to be a tiny glial mantle around neuronal components, forming a kind of “neuronal atmosphere”, for instance protecting axons once they loose their myelin sheaths (de Castro, 1937; de Castro and Herreros, 1945; Figure 3). In the ganglia the Schwann cells behave as the oligodendrocytes in the CNS, but de Castro also suggested that expansions emanated by Schwann cells form the intermediate portions of synapses, i.e., thin lamina interposed between the preganglionic fibers and the ganglionic neurons (de Castro, 1937; del Río-Hortega and Prado, 1941; de Castro, 1942; del Río-Hortega and Prado, 1942). It should be quoted here that de Castro, together with B.I. Lawrentjew, was among the first scientists specifically studying regeneration of synaptic contacts in the sympathetic system (Lawrentjew, 1925, 1934a,b; de Castro, 1930). In this series of scientific articles, de Castro showed in detail the cytoarchitecture of sympathetic and parasympathetic autonomic motor ganglia in humans, in other primates and in several large mammals. As a result of this research, de Castro was in 1924 awarded with the Martínez y Molina Prize (again from the Spanish Real Academia Nacional de Medicina). At that time the exhaustive and expert works of Fernando de Castro in the field of the histology of somatic sensory and autonomic ganglia had gained international recognition. The most clear example of this came by hand of the famous American neurosurgeon and neuropathologist Wilder S. Penfield (1891–1976), founder of the prestigious Montreal Neurological Institute (Canada): penfield invited de Castro to write two chapters for the first edition of his celebrated treaty “Penfield Cytology and Cellular Pathology of the Nervous System” (de Castro, 1932a,b). Penfield himself juicily described his “Quixotian adventure” (in his own words): his trip from the Presbyterian Hospital in New York, USA to 1924’s Madrid to work in the laboratory of Pío del Río-Hortega. In particular he describes his visit to Cajal’s laboratory on May 11th, to meet Cajal, Fernando de Castro and Domingo Sánchez:

“Cajal looked at his watch and I looked at Asúa. But at that moment, a young fellow, Fernando de Castro, came in. Cajal seemed to brighten up and said that de Castro was master of his (Cajal’s) gold method for neuroglia and suggested that I could work sometimes at a table where de Castro would teach me.

Cajal left us then and I did stay on to talk with de Castro. Dr. Sánchez insisted that I should examine with his microscope the complicated structure of an insect’s brain, explaining that the brain of an ant or a bee was just as vast in its complexity as the brain of man or any other mammal. I marveled at what he showed me and at the beautiful sections of mammalian sympathetic nerve cells on de Castro’s desk.” (Penfield, 1977).

**Figure 4:**
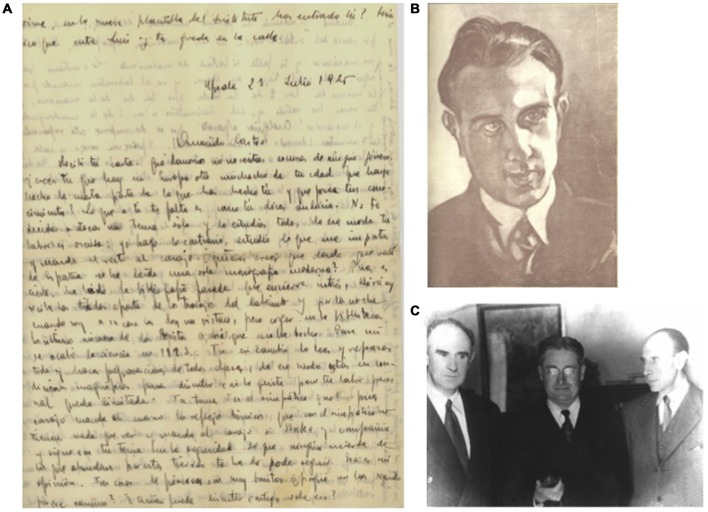
**Some proofs of the long-term friendship developed by Fernando de Castro with other disciples and visitors of the laboratory of Santiago Ramón y Cajal. (A)** Manuscript letter from Rafael Lorente de Nó to Fernando de Castro describing the excellent formation and situation of de Castro in the neuroscientific panorama of the mid 1920’s. **(B)** Original charcoal portrait of Fernando de Castro by Ferenc Miskolzy (made in 1926, in Madrid), Hungarian painter and brother of the founder of modern Hungarian Neurology, Deszo Miskolzy, disciple and translator of Cajal’s books and close friend of de Castro for years. The painter came to Spain because he wanted to visit exiled last Austro-Hungarian empress, Zita, exiled in Spain. Following recommendations of his brother Deszo, he visited Fernando de Castro at Madrid, who showed him his scientific drawings and his deep interest and knowledge in Art. The painter gifted this charbon portrait to the Spanish neuroscientist as a proof of the close frienship of both Miskolzy brothers and Fernando de Castro. **(C)** Madrid (Spain), December 1958, from the left to the right, Florencio Bustinza (1902–1982; born at Liverpool, pharmacologist and profesor of Biology at Madrid), Sir Howard Florey (Nobel Prize in Physiology or Medicine 1945) and Fernando de Castro. Bustinza was personal friend of Sir Alexander Fleming and Sir Howard Florey since 1948, and Fernando de Castro kept frienship with the latter since his time at Cajal’s laboratory to learn histological technique during the mid 1920s, **(A–C)** are part of Archive Fernando de Castro.

Because, indeed, Fernando de Castro was personally entrusted by Ramón y Cajal to direct the technical training and the research of all the fellows and researchers who arrived between 1924 and 1932 from the entire world to learn and work at the Cajal Institute, like, among many others, Deszö Miskolczy (1894–1978; considered as the father of Neuroscience in Hungary), Howard Florey (1898–1968; awarded with the Nobel Prize in Physiology or Medicine in 1945), André Dewulf (Belgium), or Clemente Estable (1894–1976; Uruguay). A number of visitors became significant friends of Fernando de Castro (Figures 4B,C). Penfield did not formally work at Cajal’s laboratory, partly due to the so vaunted distancing between the Maestro and his disciple, as Penfield himself writes:

“There was no doubt that, as I had chosen Hortega, I should continue behind him. Unfortunately, there was no extra time to work with de Castro. Hortega had spread off. The most recent discoveries came from him and his research was still far from completed. But I worked on, day by day, sitting at the desk beside Don Pío del Río-Hortega” (Penfield, 1977).

One of the most characteristic aspects of de Castro’s research is the exhaustive study of synaptic connectivity established within autonomic motor ganglia. This could be considered as his first interest in the synapse. This interest represents a continuum along the remaining of de Castro’s career.

## A Drastic Change of Direction with Consequences for de Castro’s Scientific Career

After 1925, Fernando de Castro combined his work on autonomic ganglia with the study on the innervation of the aorto-carotid region. This work fundamentally changed the field (for specific reviews in this subject, see de Castro, 2009; González et al., 2014—included in this current Special Research Topic; Figure 4A). In the attempts to prove his hypothesis that neurons located in the carotid bodies act as sensory chemoreceptors that detect changes in the chemical composition of circulating blood, Fernando de Castro took an alembicated experimental way that should pass through… the orthosympathetic ganglia! Lesions of the superior cervical ganglion were instrumental to study degeneration and regeneration of the intra-ganglionic synapses in the carotid bodies (de Castro, 1930), as an easier and indispensable step to attack regeneration of “his” aorto-carotid fibers in the future. The 1929 article represents a prelude of a full reorientation in de Castro’s research during the decade starting in 1930: in agreement with Cajal’s advice, Fernando de Castro had begun studying neural tissue *in vitro* and regeneration of the nervous system in collaboration with the un-discussed leader of the field at that time, Giusseppe Levi (1872–1965). De Castro spent several periods in Levi’s laboratory. These postdoctoral fellowships had the potential to be very successful for Fernando de Castro. However, political events (Giuseppe Levi was imprisoned by the fascist Mussolini government after a problem between Levi’s son and the police) and unexpected health problems of the young Spanish neurohistologist (for details in this novelistic episode, see de Castro, 2009; Santarén and Sánchez-Ron, 2009) seriously limited the planned research. The direct and indirect results of the experiments undertaken in the Italian collaboration became incorporated in work published in the immediate years after de Castro had returned to Madrid (de Castro, 1934, 1937).

In this period, de Castro’s colleague and friend, Rafael Lorente de Nó (1902–1990; together with de Castro, the last and youngest direct disciples of Cajal) strongly advised Ramon y Cajal that de Castro should never abandon the study of the aorto-carotid innervation (de Castro, 1972, 2009; Santarén, 2014). While Lorente’s advice was correct, the decision by de Castro and Cajal to continue the aorto-carotid innervation research line may be considered in the light of world history to have had a negative impact on to de Castro’s scientific career. Civil war erupted in Spain in 1936 and fighting reached Madrid towards the end of that year. Fernando de Castro, being in charge of protecting the equipment and collections of the Cajal Institute, became fully occupied in protecting the Institute from literal disappearance during the almost 3 years (1936–1939) that the Spanish Civil War ravaged Madrid (de Castro, 2009; De Carlos and Pedraza, 2014; González et al., 2014). In the mean time the Belgian physio-pharmacologist Corneille Heymans (1892–1968) took advantage of the opportunity and won the race to functionally demonstrate the origin in the carotid body of the chemical reflexes. Heymans consequently was awarded in 1938 with the Nobel Prize in Physiology or Medicine.

## Post-War Studies on the Synaptic Organization of the Sympathetic Ganglia

**Figure 5:**
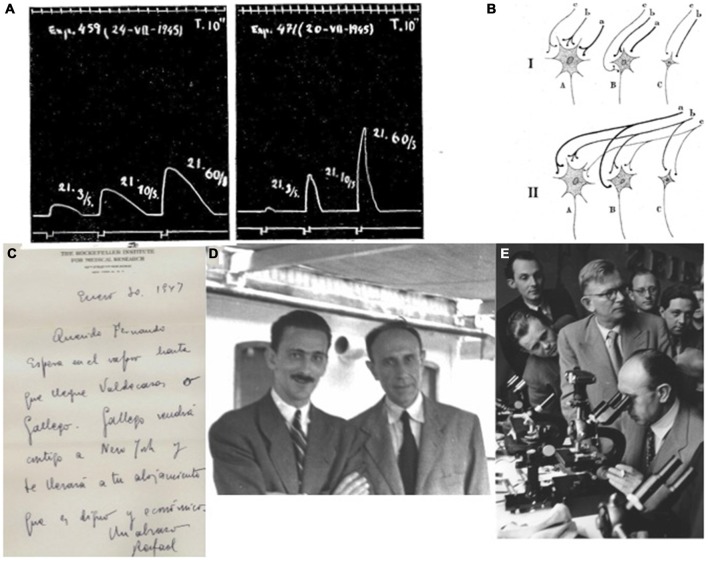
**Post-war de Castro on the autonomic nervous system and their synaptic structure. (A)** Electromiographic recordings of the nictitant membrane of the adult cat where the sympathetic superior cervical ganglion has been innervated by rami from the VI-c and VII-c nerves (de Castro and Herreros, 1945). This 10 s-recording shows that the intensity of the contraction correlates with the frequency of the tetanic stimulation. **(B)** Schematic representation of the preganglionic convergence of fibers (*a*, *b*, *c*) onto ganglionic cell types (*A*, *B*, *C*). The thickness of the fibers is representative of their thickness *in vivo*. In this it is assumed that ganglion cells can trigger when activated by two boutons simultaneously, or in the slow fibers (*c*) when a sinchronic impulse via a-b facilitates it (de Castro and Herreros, 1945). **(C)** Original letter from Rafael Lorente de Nó to Fernando de Castro (dated at the Rockefeller Institute, New York on January 30th, 1947): *“Dear Fernando, Wait at the shipboat till the arrival of Valdecasas or Gallego. Gallego will come with you to New York and will bring you to your accomodation, that is worthy and economic. Hugs, Rafael”* (translated by the autor of this work from Spanish). **(D)** Antonio Gallego (1915–1992) and Fernando de Castro on board of the *Motomar* steamship, in their way back to from New York to Spain (1947) after their respective first scientific experience at the USA. **(E)** Fernando de Castro (at the microscope), invited speaker to expose the cytoarchitecture of the autonomic nervous system. Became one of the main characters in the final offical defeat of reticularists at the 34 Tagung Deutschen Gesselchaft fü Pathologie (Wiesbaden, Germany; 1950). His friend, the German histologist established in Chile, Emil Herzog (on foot, with glasses, just behind Fernando de Castro) acts as de Castro’s master of ceremony at that time, **(A–E)** are part of Archive Fernando de Castro.

Times changed and Fernando de Castro decided to attack one of his scientific dreams, postponed for years due to the Spanish Civil War (1936–1939) and the Second World War (1939–1945). The study of structure and function of synapses had significantly progressed in these years already, and de Castro assumed that the study of synapses in the autonomic nervous system would really be profiting since the structure of the ganglia is simpler than that of the CNS and his particular knowledge on the fine structure of the sympathetic ganglia would undoubtedly be of great help in this new research (Figures 5A,B). As soon as the political circumstances permitted, de Castro contacted his old friend Rafael Lorente de Nó who had emigrated to the USA in 1931 to fulfill a fellowship at the Rockefeller University in New York. Fernando de Castro’s travel request to the USA was accepted and granted by the Junta de Relaciones Culturales (Spain), de Castro arrived in New York at the beginning of 1947, to work with Herbert S. Gasser (Nobel Prize in Medicine or Physiology on 1944, shared with Joseph Erlanger) and Lorente de Nó and to learn the basics of electrophysiology and electrophysiological recordings (Figures 5C,D).

In these years, de Castro insisted on the fact that is the protoplasmic glia that is interposed between the pre- and the postsynaptic elements (de Castro, 1942, 1951). De Castro showed that pericellular nests are the way through which presynaptic fibers contact postsynaptic neurons, although these nests appear to be larger and more frequent in Amphibians and Reptiles than in Mammals. According to de Castro there are more synapses than morphologically identifiable terminal boutons in sympathetic ganglia, as he demonstrated by sectioning preganglionic fibers (de Castro and Herreros, 1945). This drove him to propose that preganglionic fibers form in these autonomic ganglia a kind of diffuse connection beyond the terminal boutons (de Castro, 1951).

## Concluding Remarks

The work by Fernando de Castro to get his PhD degree at the beginning of the 1920’s decade produced capital ammunition to destroy the reticularist conception of the organization of somatic sensory and autonomic nervous ganglia (Figure 5E). De Castro described the delicate morphological details of the ganglionic cells and the distribution of the synaptic connections in such a meticulous and convincing way that it revolved the field. De Castro’s work in this field fully granted him the technical and intellectual recognition by his tutor, Santiago Ramón y Cajal, and it prepared him for the study of the innervation of blood vessels, particularly those in the carotid region, to identify the controversial nature of this innervation triggering the cardio-respiratory reflexes. He was the first to identify arterial chemoreceptors in the carotid bodies. After the forced break due to both the Spanish Civil War and the Second World War, Fernando de Castro continued working on sympathetic ganglia to study synapses and synaptogenesis. For the rest of his scientific career till his death in 1967, both the arterial chemoreceptors and the autonomic and somatic sensory ganglia remained his principal research lines. His histological descriptions remain fully recognized and actual today.

## Author Contributions

The author confirms being the sole contributor of this work and approved it for publication.

## Conflict of Interest Statement

The author declares that the research was conducted in the absence of any commercial or financial relationships that could be construed as a potential conflict of interest.
